# The complete mitochondrial genome of *Periacma orthiodes* Meyrick, 1894 (Lepidoptera: Autostichidae)

**DOI:** 10.1080/23802359.2021.1960217

**Published:** 2021-08-04

**Authors:** Aihui Yin

**Affiliations:** School of Basic Medicine, Guizhou University of Traditional Chinese Medicine, Guiyang, Guizhou, China

**Keywords:** *Periacma orthiodes*, mitochondrial genome, Autostichidae, phylogenetic analysis

## Abstract

The complete mitogenome of *Periacma orthiodes* Meyrick, 1894 was determined in this study. It was 15,306 bps long and strongly AT biased. It consisted of 13 PCGs, 22 tRNAs, 2 rRNAs and 1 non-coding control region (371 bps). Most PCGs used the typical ATN start codon, except for *cox1*. Four genes (*cox1*, *cox2*, *nad4* and *nad5*) used truncated stop codons (a single T or TA) rather than the commonly used TAA or TAG. All tRNAs, excluding TrnS1, folded into the iconic cloverleaf structure. ML phylogenetic tree built on 13 PCGs from *P. orthiodes* and another 28 species in Gelechioidea demonstrated that the genus *Periacma* was a member of the family Autostichidae, which was consistent with the newest phylogenetic study.

*Periacma* Meyrick, 1894 is a medium-sized microlepidopteran genus with more than 80 described species, and mainly distributed in the Oriental and Palearctic Regions. *Periacma* with three other closely allied genera: *Epiracma*, *Irepacma* and *Ripeacma* (including more than 60 species altogether), constitutes a relatively derived group within the superfamily Gelechioidea, by sharing the peculiar synapomorphic character that the males have two-segmented labial palpus (Lvovsky 2005). However, due to the lack of analytical study, the taxonomic status of this genus group has long been obscure (Kim et al. 2016). Hence, these genera have been placed in different families or subfamilies more of less based on the intuitions of different authors (e.g. Lvovsky 2005; Wang and Li 2006; Lvovsky 2012). In recent years, several molecular phylogenetic studies concerning these genera were successively conducted, whereas, with inconsistent results. In Kim et al. (2016), these genera were attributed in the family Xyloryctidae; but in Wang and Li (2020), they were recovered as members of the family Autostichidae. The latter result was supported by a mitogene-based, though very limitedly sampled phylogenetic analysis (Zhi and Yin 2021). Thus, in the present study, the author determined the first complete mitogenome of *Periacma*, the most speciose and representative genus of the group, and then used it to further verify the systematic position of this genus group. The mitogenome was from *Periacma orthiodes* Meyrick, 1894, a common species from Oriental Region. The adult of *P. orthiodes* was collected from Maolan Natural Reserve (25°17'10"N, 108°42'42"E), Guizhou, China in 2020, using light trap, then preserved in pure alcohol before sent for sequencing.

The NGS data were generated using the PE 150 sequencing method on the Illumina NovaSeq 6000 platform by Novogene (Tianjin, China). The full length mitogenome was de novo assembled by MitoZ V.2.3 (Meng et al. 2019) and SPAdes V.3.15.1 (Bankevich et al. 2012). Sequence polish was aided with BWA V.0.7.17 (Li 2013), samtools V.1.7 (Li et al. 2009) and Pilon V.1.23 (Walker et al. 2014). Both MitoZ software and MITOS Web Server (http://mitos2.bioinf.uni-leipzig.de/index.py) were utilized for annotation. The remaining specimen tissue and the extracted DNA were deposited under −20 °C in the Insect Collection of Guizhou University of Traditional Chinese Medicine, Guiyang, China (Aihui Yin, keyanlaodong@163.com, Voucher specimen: GZUTCM:M10).

Our sequencing data assembled a complete circular mitochondrial sequence of 15,306 bps in length (GenBank: MW697075). It was comprised of 13 PCGs, 22 tRNA genes and 2 rRNA genes. Overall base composition of the mitogenome was A: 37.8%, T: 42.6%, C: 11.8%, G: 7.8%. AT contents of PCGs, tRNAs, and rRNAs of *P. orthiodes* were 78.8%, 81.6%, and 84.7%, respectively. All PCGs of *P. orthiodes* started at the codon of ATN, except for *cox1* with unorthodox CGA as the start codon. Four PCGs of *P. orthiodes* ended in truncated stop codons (*cox1*, *cox2*: T; *nad4* and *nad5*: TA). The rest nine PCGs used TAA or TAG as stop codons. All tRNAs could fold into the iconic clover-leaf secondary structure, except for TrnS1, which formed a single stranded loop instead of the DHU arm. The special tRNA gene order TrnM-TrnI-TrnQ, which existed extensively in the mitogenomes of the Ditrysian moths was also observed in *P. orthiodes* (Cao et al. 2012; Park et al. 2016). The putative A + T rich control region was 371 bps in length, and had very high AT content (94.1%), remarkably higher than that of the whole mitogenome (80.4%), PCGs, tRNAs or rRNAs.

Thirteen concatenated PCGs of *P. orthiodes*, plus all 28 Gelechioidea mitogenomes with the PCG parts fully sequenced available from GenBank were used to reconstruct the ML phylogenetic tree via IQTREE V.2.07 (Nguyen et al. 2015; Figure 1). Two species from the family Tortricidae were chosen as outgroups. The partitioning scheme for the three codon positions of the 13 genes was determined by the TESTMERGE option in IQTREE. Seven partitions were finally created and applied with their own best fit substitution model and parameters (GTR + F + I + G4, K3Pu + F + I + G4, GTR + F + I + G4, TIM2 + F + I + G4, TIM3 + F + G4, GTR + F + I + G4, K3Pu + F + I + G4). The standard bootstrap analysis (1000 pseudoreplicates) was executed to produce the branch support values (BSVs). The tree topology suggested that all the families with multiple representatives, except for Oecophoridae, were recovered as monophyla. Autostichidae was sister to Xyloryctidae. *P. orthiodes* was doubtlessly a member of Autostichidae, only it clustered in the same branch with *Meleonoma mirabilis* rather than the presumed *Ripeacma umbellata* (BSV = 64).

**Figure 1.**
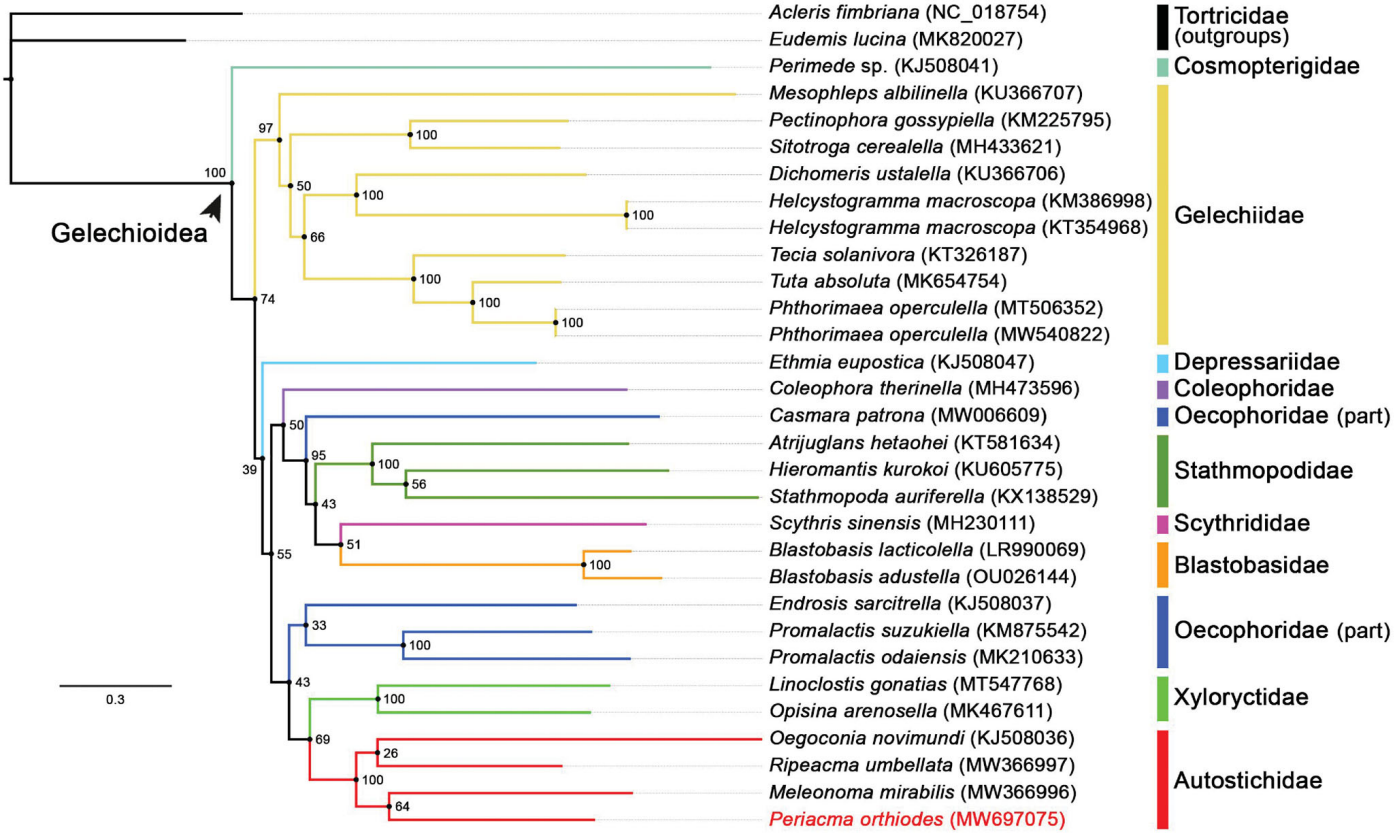
ML tree showed phylogenetic relationships within the superfamily Gelechioidea (*P. orthiodes* in red). BSVs were labeled at tree nodes and GenBank accession numbers were in parentheses.

## Data Availability

The genome sequence data that support the findings of this study are openly available in GenBank of NCBI at https://www.ncbi.nlm.nih.gov/nuccore/MW697075 under the Accession no. MW697075. The associated BioProject, SRA, and Bio-Sample numbers are PRJNA737313, SRR14804793 and SAMN19689793, respectively.
